# A Diagnostic Method for Gastric Cancer Using Two-Photon Microscopy With Enzyme-Selective Fluorescent Probes: A Pilot Study

**DOI:** 10.3389/fonc.2021.634219

**Published:** 2021-08-27

**Authors:** Choong-Kyun Noh, Chang Su Lim, Gil Ho Lee, Myung Ki Cho, Hyo Won Lee, Jin Roh, Young Bae Kim, Eunyoung Lee, Bumhee Park, Hwan Myung Kim, Sung Jae Shin

**Affiliations:** ^1^Department of Gastroenterology, Ajou University School of Medicine, Suwon, South Korea; ^2^Department of Energy Systems Research and Department of Chemistry, Ajou University, Suwon, South Korea; ^3^Department of Pathology, Ajou University School of Medicine, Suwon, South Korea; ^4^Office of Biostatistics, Ajou Research Institute for Innovation Medicine, Ajou University Medical Center, Suwon, South Korea

**Keywords:** gastric cancer, diagnosis, ratiometric fluorescent probes, two-photon fluorescent imaging, endoscopy

## Abstract

**Background:**

Endoscopy is the most important tool for gastric cancer diagnosis. However, it relies on naked-eye evaluation by endoscopists, and the histopathologic confirmation is time-consuming. We aimed to visualize and measure the activity of various enzymes through two-photon microscopy (TPM) using fluorescent probes and assess its diagnostic potential in gastric cancer.

**Methods:**

β-Galactosidase (β-gal), carboxylesterase (CES), and human NAD(P)H: quinone oxidoreductase (hNQO1) enzyme activities in the normal mucosa, ulcer, adenoma, and gastric cancer biopsy samples were measured using two-photon enzyme probes. The fluorescence emission ratio at long and short wavelengths (Ch2/Ch1) for each probe was comparatively analyzed. Approximately 8,000 – 9,000 sectional images in each group were obtained by measuring the Ch2/Ch1 ratio according to the tissue depth. Each probe was cross-validated by measuring enzymatic activity from a solution containing lysed tissue.

**Results:**

Total of 76 subjects were enrolled in this pilot study (normal 21, ulcer 18, adenoma 17, and cancer 20 patients, respectively). There were significant differences in the mean ratio values of β-gal (0.656 ± 0.142 *vs*. 1.127 ± 0.109, *P* < 0.001) and CES (0.876 ± 0.049 *vs*. 0.579 ± 0.089, *P* < 0.001) between the normal and cancer, respectively. The mean ratio value of cancer tissues was different compared to ulcer and adenoma (*P* < 0.001). The hNQO1 activity showed no significant difference between cancer and other conditions. Normal mucosa and cancer were visually and quantitatively distinguished through β-gal and CES analyses using TPM images, and enzymatic activity according to depth, was determined using sectional TPM ratiometric images. The results obtained from lysis buffer-treated tissue were consistent with TPM results.

**Conclusions:**

TPM imaging using ratiometric fluorescent probes enabled the discrimination of gastric cancer from normal, ulcer, and adenoma. This novel method can help in a visual differentiation and provide quantitative depth profiling in gastric cancer diagnosis.

## Introduction

Upper endoscopy is an important screening and diagnostic tool for gastric cancer (1–3). Currently, the standard process for diagnosing gastric cancer is performing an upper endoscopy, collecting tissue samples from the lesion, and obtaining histopathologic confirmation (4). However, this process cannot be performed in real-time and is time-consuming. Moreover, inter-observer discrepancies in pathologic evaluation may occur (5).

Determining the feasibility of endoscopic resection, such as endoscopic submucosal dissection (ESD), depends on the judgment of the endoscopist. To overcome these limitations, various assistive methods (chromoendoscopy, image-enhanced endoscopy, and confocal microscopy) have been developed (6–9). However, inter-observer discrepancy still occurs with these methods, and depth and margin evaluations rely on the endoscopists’ experience and decision.

Methods for visualizing lesions using fluorescence imaging with various probes through two-photon microscopy (TPM) have been developed (10, 11). These probes induce enzymatic reactions through fluorescence activation. TPM uses two near-infrared photons for excitation to minimize autofluorescence generated within tissues and creates images of sections between the surface and deeper tissue layers (12–14). Using these images, TPM can generate real-time high-resolution 3-dimensional (3D) images, and probe photobleaching and photodamage are remarkably minimalized compared to confocal microscopy because a femtosecond pulse laser is used. This strategy allows for precise lesion analysis according to depth by producing multiple sectional images along the z-direction from thick tissue samples (15).

We evaluated various biomedical applications of TPM, including in cancer diagnosis in previous studies (16–18). We showed that human NAD(P)H: quinone oxidoreductase (hNQO1) activity gradually increased in normal, adenoma, and adenocarcinoma colon tissues through ratiometric TPM imaging, and reported the depth through imaging (16). We hypothesized that the ratiometric probes that were confirmed through TPM could be used for gastric cancer diagnosis. We also supposed that the acidic stomach environment could affect the enzymatic reaction of the fluorescent ratiometric probes, and would differ from the colon study results.

In this study, we evaluated probes applicable for gastric cancer diagnosis and assessed their enzyme detection ability in gastric cancer tissues with TPM. We also examined lesion depth using these probes and validated candidate probes using samples treated with lysis buffer. These findings may lead to a faster, more reliable diagnosis of gastric cancer.

## Materials and Methods

### Patients

**Figure 1:**
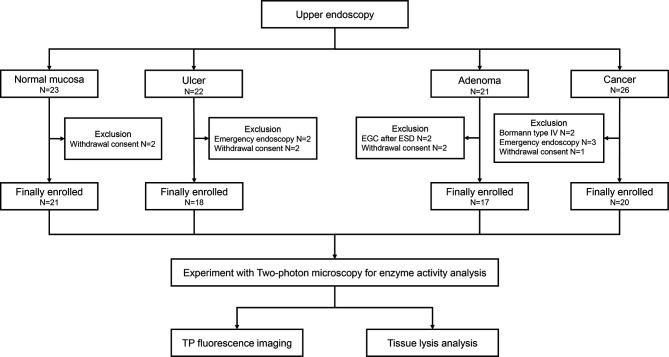
Flow diagram for selection of the study population.

The present study was a prospective study conducted at the Ajou University Hospital (Suwon, Republic of Korea) between March 2019 and October 2019. A total of 20 patients who were diagnosed with gastric cancer were enrolled in this study. We also enrolled patients with gastric ulcer, gastric adenoma, and healthy individuals to compare with cancer (**Figure 1**). Bormann type IV advanced gastric cancer, inoperable cases, and cases requiring emergency endoscopy due to bleeding or obstruction were excluded. The study protocol was approved by Ajou University Hospital Institutional Review Board (approval no. AJIRB-BMR-SMP-18-373). Written informed consent of all patients was obtained prior to study participation.

### Pathologic and TPM Evaluation After Specimen Acquisition

After upper endoscopic assessment of the lesion, three tissue samples were collected from the lesion, placed in containers with saline gauze, and transported to the Department of Chemistry for further analyses. For pathologic confirmation, four additional samples were collected from the lesion. In healthy individuals, seven samples (normal mucosa) were collected and used for TPM and pathologic evaluation. All samples were fixed in 10% buffered formalin solution and embedded in paraffin. A standard histopathological process including hematoxylin and eosin staining, was conducted.

### Two-Photon Probes for Enzymatic Activity Measurement

**Figure 2:**
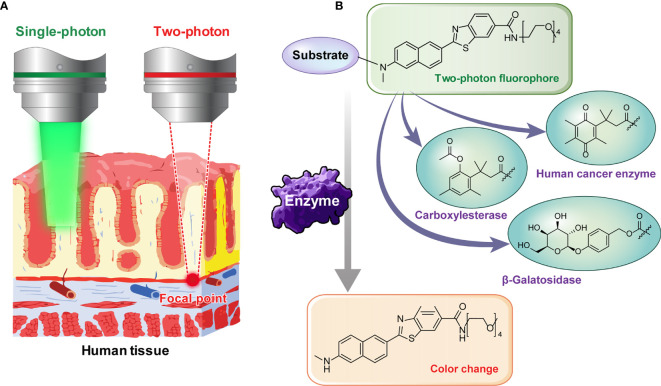
**(A)** Illustration showing the necessity of two-photon microscopy for deep tissue imaging. **(B)** Image showing two-photon probes that change color as the substrate is removed by reacting with various enzymes.

**Figure 3:**
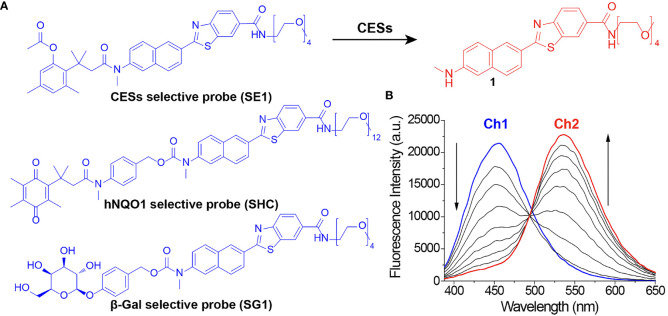
**(A)** Chemical structure of two-photon probes and **(B)** fluorescence spectrum change in SE1 because of structural changes.

There has always been limitations to image acquisition from the specific surface of a tissue using conventional confocal microscope. To overcome this and obtain an image from deep part of the tissue, TPM can be used. By using a TP excitation source that utilizes low energy photons, it becomes possible to obtain high-quality images of the specific desired layer, deep in the tissue. Early diagnosis will be possible if the presence or absence of a lesion deep in the human tissue specimen is confirmed by utilizing these advantages. To use TPM, development of a suitable TP probe must also be supported. The probes that we used in this study do not only show fluorescence by selectively responding to a TP light source but also have the property of changing fluorescence color in response to specific enzymes. We applied probes that react selectively to the cancer related enzymes. This is possible by using TP phosphor as a basic skeleton that responds well to a two-photon light source and emits the fluorescence effectively (**Figure 2**). Enzymes known to display disproportionate expression in association with cancer progression (β-galactosidase [β-gal], carboxylesterase [CES], and human NAD(P)H: quinone oxidoreductase [hNQO1]) (19–21) were selected as detection targets for gastric cancer diagnosis. This study was conducted using three probes, SG1 (15), SE1 (17), and SHC (16), which enable effective observation of the three enzymes mentioned above, respectively. The two-photon (TP) probe structure consisted of a substrate that reacted with the target enzyme and fluorophore that acted as the TP dye. PEG unit were introduced as the solubilizing unit in buffer to enhance the cell loading ability. As each probe reacted with the target enzyme, the substrate separated from the basic fluorophore skeletal structure, as shown in **Figure 3**. Additionally, each probe was designed to change its fluorescence wavelength from short (Ch1) to long (Ch2) as it reacted with the target enzyme and undergo structural changes to generate a fluorophore. Owing to this characteristic, all three probes display a larger Ch2/Ch1 value as the enzymatic activity becomes stronger. As a result, enzyme level changes can be accurately analyzed using the Ch2/Ch1 ratio calculated after measuring fluorescence intensity from the images obtained from the two fluorescent regions. In our previous study, each probe was used the same way to produce similar results (16–18, 22).

### TP Fluorescence Diagnostic Process in Stomach Tissues

**Figure 4:**
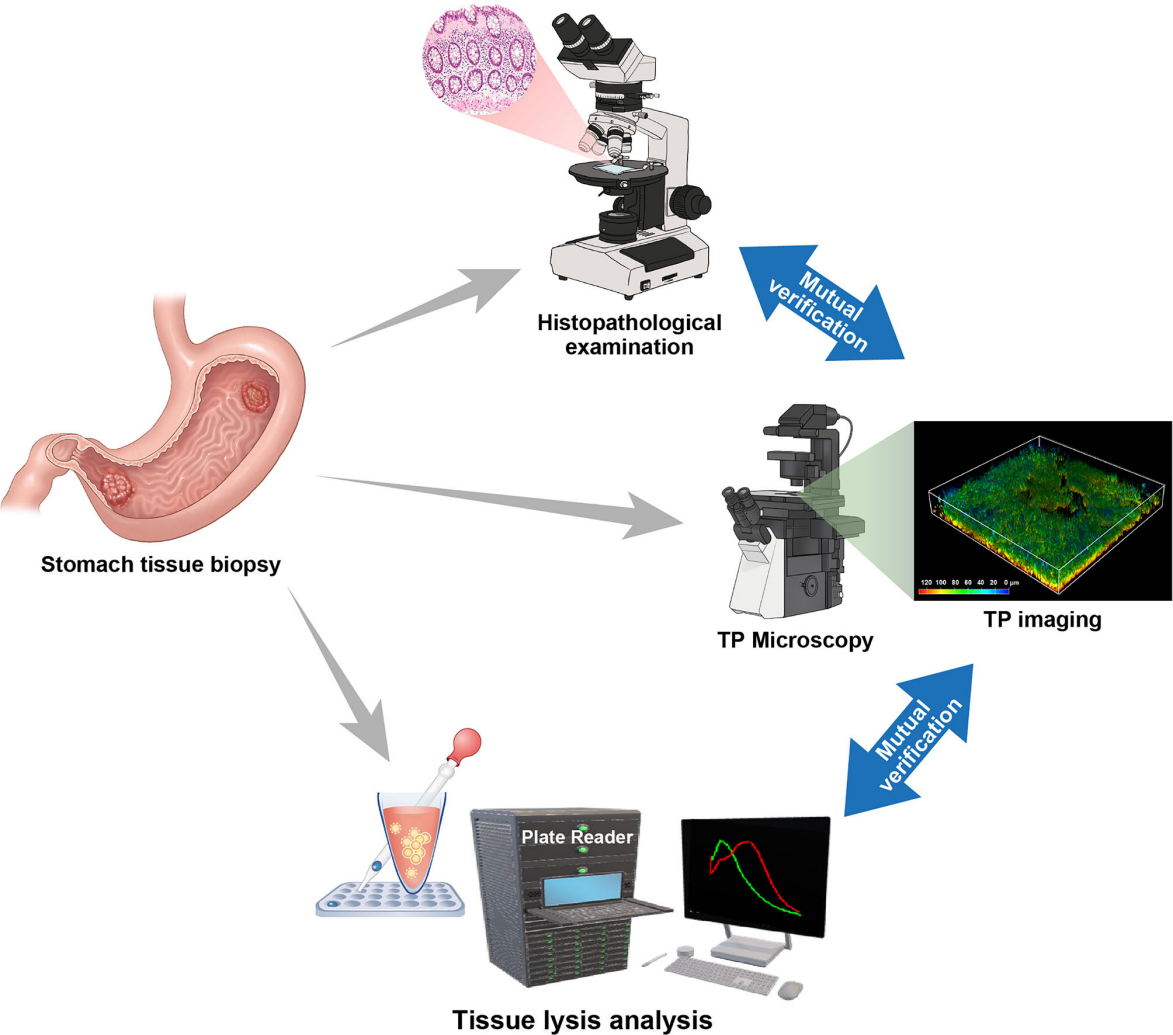
Two-photon fluorescence imaging through the acquisition of stomach tissue specimens using a mutual verification system including histological examination and tissue lysis fluorescence analysis for an accurate diagnosis.

To establish an imaging procedure for cancer diagnosis using stomach tissue, the TP probes were applied to patient tissue specimens. The tissue specimens were then divided for TP fluorescence imaging and tissue lysis analysis (**Figure 4**). The specimens for fluorescence imaging were further divided into three groups for each patient. First, the TP probes were used to stain normal and gastric cancer tissues for one hour before TP fluorescence imaging was conducted. From the fluorescence images generated by each probe, TP fluorescence imaging was conducted according to target tissue depth. The Ch2/Ch1 fluorescence ratio obtained as each probe reacted with its target enzyme at different depths was used to analyze enzymatic activity (22). Imaging and tissue lysis analyses were performed simultaneously, and two expert pathologists conducted and peer reviewed the histopathological evaluation to compare the results. Through these processes, the feasibility of accurate diagnosis by TP fluorescence imaging was examined. The imaging process took approximately two hours to analyze the enzymatic activity from each depth through imaging and to obtain the final results enabling diagnosis.

### TPM Imaging of Enzymatic Activity in Stomach Tissues

**Figure 5:**
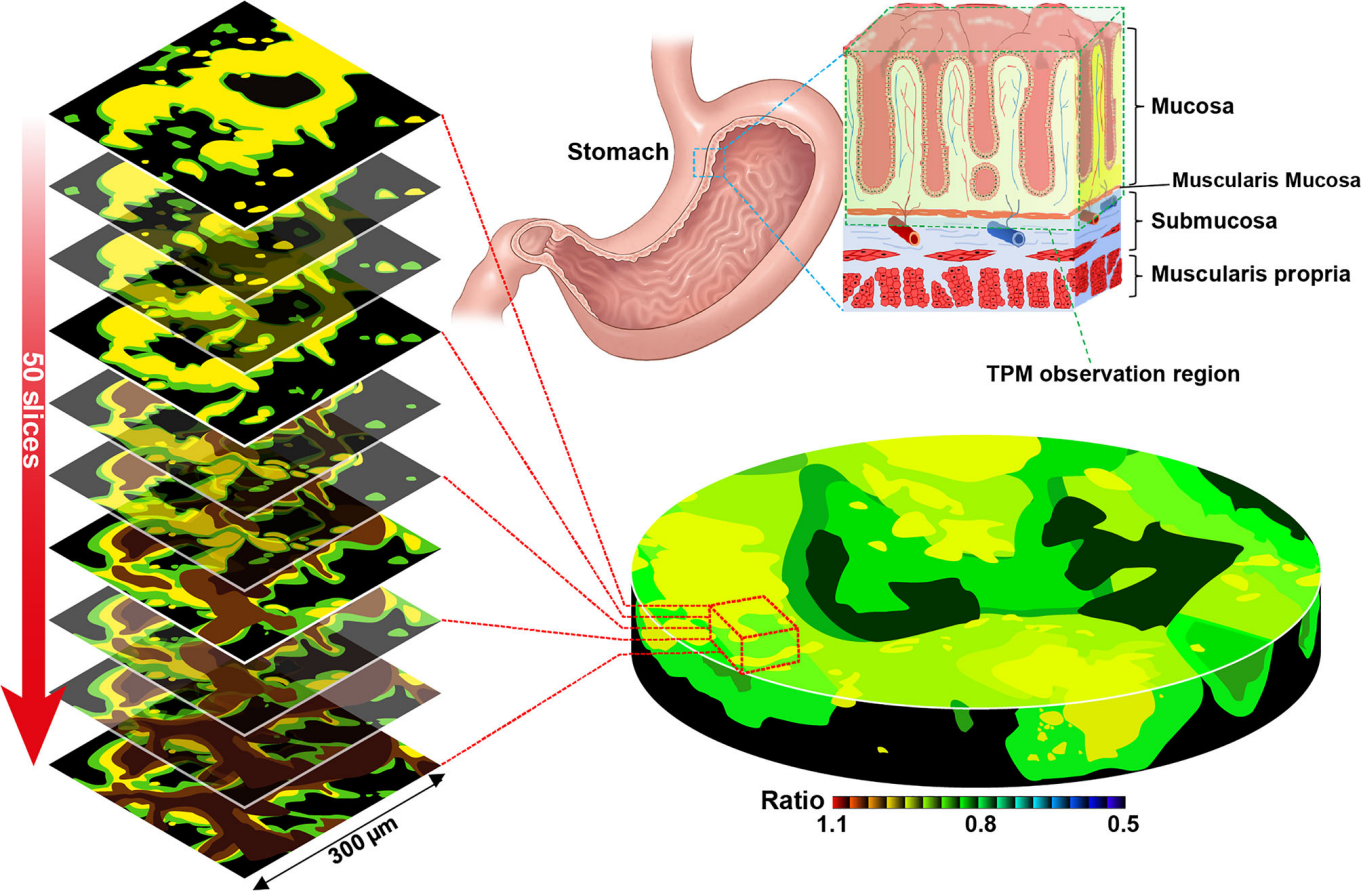
Two-photon microscopy sectional ratiometric image of the area indicated by the red box and 3-dimensional ratiometric two-photon microscopy image of stomach tissue with SE1 (Schematic illustration).

Tissue samples were collected from each patient and immediately stained with 10 μM TP probes. The enzyme level and pH changes of the target tissues according to lesion depth were measured by TPM. We also measured enzymatic activity at each depth using the Ch2/Ch1 ratio from the surface to deep (90–210 μm) inside the tissue sample (**Figure 5**). Experiments were performed using patient biopsy samples containing mucosal to submucosal stomach tissue. In the actual imaging experiments, the mucosa was placed facing the bottom of the imaging dish and then imaged. Each sectional image was obtained using two fluorescence regions (Ch1; 400**–**450 nm and Ch2; 600**–**650 nm, **Figure 3B**) and converted to a 3D TPM ratiometric image. In order to analyze the enzyme activity in normal and cancer tissues labeled with the probe, approximately 8000–9000 sectional images were used in each group. In addition, enzymatic activity was comparatively analyzed using the mean intensity ratio values obtained from the images using the entire sample depth. Additionally, no fluorescence was observed in the experiment conducted without TP probes under the same imaging conditions, and the possibility of error in the experiment due to self-fluorescence could be excluded (**Supplementary Figure S1**).

### Enzyme Activity Analysis With Ratio Values After Homogenization

**Figure 6:**
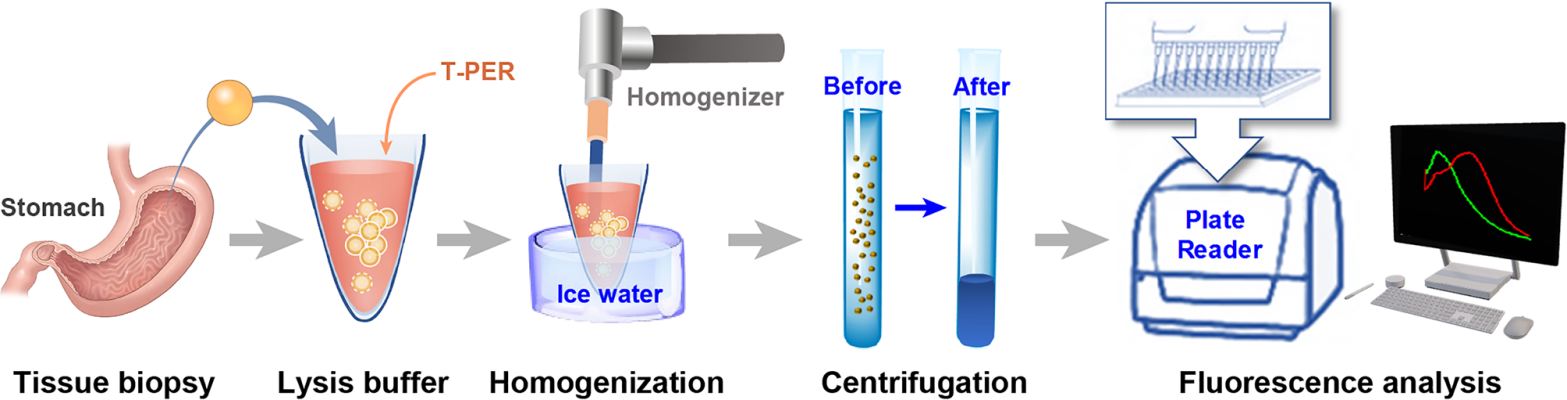
The Process of enzyme activity analysis using tissue lysis solution.

We measured and compared enzyme activity in lysis solutions of normal and cancerous tissues for cross validation of imaging diagnostics (**Figure 6**). In this experiment, sample tissue was dissolved using T-PER (Tissue protein extraction reagent, Pierce, Rockford, IL, USA) and pulverized using a tissue homogenizer after cooling to 0°C with ice water to prevent protein modification. Then, the lysate was centrifuged at 15,000 rpm at 4°C for 5 min to obtain a solution containing the enzyme to be detected. The separated solution was quantified to 200 μg of protein using total protein levels measured using a bicinchoninic acid protein assay reagent kit (Gen DEPOT, Katy, TX, USA). Then, each 1 μM probe was processed for fluorescence analysis using a plate reader (Varioskan Flash, Thermo Fisher Scientific, Waltham, MA, USA). The reactions between each probe and enzyme were monitored every 10 min for a total of 120 min and the altered fluorescence intensity ratios of short and long wavelengths (Ch2/Ch1) were used to analyze the differences in enzymatic activity. Spectra were acquired at 0 to 100 min after the addition of probes with a multi-detection microplate reader (Varioskan Flash).

### Western Blot Analysis

We carried out western blot experiments to validate the three enzymes. Specific details of the method are described in the **Supplementary Material**.

### Statistical Analysis

The primary outcome was to determine probes’ applicability for gastric cancer diagnosis and to assess their enzyme detection ability in gastric cancer tissues through TPM. Our secondary outcome was to evaluate lesion depth using these probes and to compare cancer with other benign conditions, such as ulcer and adenoma. Continuous variables were expressed as the mean ± standard deviation, and categorical variables were expressed as both total numbers and percentages. The student t-test or Wilcoxon rank-sum test, as appropriate, was used to compare continuous data. All reported *P*-values were two-sided, and *P* < 0.05 was considered significant. Analyses were performed using SAS version 9.4 (SAS Institute, Cary, NC, USA).

## Results

### Patients and Baseline Characteristics

Total of 76 subjects (normal; N= 21, ulcer; N = 18, adenoma; N = 17, and cancer; N = 20, respectively) were enrolled in this pilot study (**Figure 1**). The baseline characteristics of the enrolled patients are shown in **Table 1**. Bormann type II advanced gastric cancer (8/20, 40.0%) was the most prevalent type in cancer patients. Eight patients (8/20, 40.0%) had differentiated type cancer, and pT3 (7/20, 35.0%) was the most common tumor depth. In the adenoma group, 14 patients (14/17, 82.4%) were confirmed as low-grade adenoma after endoscopic resection. A representative western blot analysis of candidate proteins is shown in **Supplementary Figure S1**. β-gal expression was enhanced in gastric cancer compared to normal tissue. CES expression was reduced in gastric cancer tissue when compared with that in normal tissue. However, hNQO1 expression was not different between cancer and normal tissue.

**Table 1:** Demographics and pathologic characteristics of enrolled patients.

Variables	Normal	Ulcer	Adenoma	Cancer
	N = 21	N = 18	N = 17	N = 20
Gender, N (%)				
Male	15 (71.4)	13 (72.2)	12 (70.6)	14 (70.0)
Female	6 (28.6)	5 (27.8)	5 (29.4)	6 (30.0)
Age, years				
Mean ± SD	66.3 ± 10.8	65.4 ± 13.4	63.1 ± 10.2	65.7 ± 10.6
BMI, kg/m^2^, mean ± SD	22.9 ± 2.6	23.7 ± 2.1	24.9 ± 2.3	23.1 ± 2.6
lesion size, mm, mean ± SD		20.8 ± 5.8	18.9 ± 5.4	45.6 ± 20.3
Lesion location, N (%)				
Upper 1/3		1 (5.6)	0 (0)	2 (10.0)
Middle 1/3		4 (22.2)	9 (52.9)	8 (40.0)
Lower 1/3		13 (72.2)	8 (47.1)	10 (50.0)
Morphology, N (%)				
Early gastric cancer				5 (25.0)
AGC Bormann type I				1 (5.0)
AGC Bormann type II				8 (40.0)
AGC Bormann type III				6 (30.0)
AGC Bormann type IV				0 (0)
Histology, N (%)				
Differentiated				8 (40.0)
Undifferentiated				12 (60.0)
Low-grade adenoma			14 (82.4)	
High-grade adenoma			3 (17.6)	
Depth of tumor invasion, N (%)				
pT1				5 (25.0)
pT2				3 (15.0)
pT3				7 (35.0)
pT4				5 (25.0)
AJCC/UICC stage, N (%)				
I				6 (30.0)
II				8 (40.0)
III				6 (30.0)
IV				0 (0)
Treatment, N (%)				
Subtotal gastrectomy				13 (65.0)
Total gastrectomy				4 (20.0)
Endoscopic submucosal dissection			17 (100.0)	3 (15.0)

Tumor stage according to the American Joint Committee on Cancer, 7th Edition.

AGC, Advanced gastric cancer; AJCC, American Joint Committee on Cancer; BMI, body mass index; SD, standard deviation; UICC, Union for International Cancer Control.

### TPM Imaging of Candidate Probes for Gastric Cancer Detection

**Figure 7:**
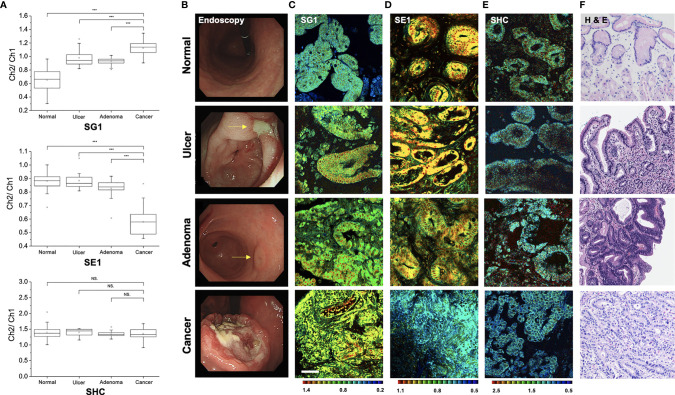
Pseudo-colored ratiometric two-photon microscopy images (Ch2/Ch1) of normal, ulcer, adenoma, and cancer tissue. **(A)** Ratio value (Ch2/Ch1) box plots of normal, ulcer, adenoma, and cancer tissue (***P < 0.001, NS, not significant). **(B)** Endoscopic image. **(C)** Two-photon microscopy images in SG1. **(D)** Two-photon microscopy images in SE1. **(E)** Two-photon microscopy images in SHC. **(F)** Histopathologic image with hematoxylin and eosin staining.

To determine the feasibility of gastric cancer diagnosis using a TP fluorescent probe, TPM imaging was conducted using the measurement conditions required for each probe (**Figure 7A**). When using SG1, which reflects β-gal activity (15), the ratio value was higher in gastric cancer tissues than in normal tissues (1.127 ± 0.109 *vs*. 0.656 ± 0.142, *P* < 0.001), gastric ulcer (0.992 ± 0.119, *P* < 0.001), and gastric adenoma (0.927 ± 0.049, *P* < 0.001). In comparison with normal samples, the ratio value of cancer was approximately 1.72-fold higher than that of the normal tissue. In contrast, the ratio value obtained using SE1, which measures CES (17), was lower in gastric cancer tissues than in normal tissues (0.579 ± 0.089 *vs*. 0.876 ± 0.049, *P* < 0.001), ulcer (0.884 ± 0.048, *P* < 0.001), and gastric adenoma (0.879 ± 0.059, *P* < 0.001). However, the ratio value obtained using SHC (16) to detect hNQO1 activity did not differ between cancer vs. normal tissue (1.354 ± 0.174 *vs*. 1.393 ± 0.141, *P* = 0.352), *vs*. ulcer (1.317 ± 0.120, *P* = 0.195), and *vs*. adenoma (1.343 ± 0.073, *P* = 0.676). Thus, SG1 and SE1 may be used to differentiate gastric cancer and other conditions.

When the ratio value between normal and cancer significantly differed, visual differentiation using TPM images was possible through fluorescence color variance. Endoscopic, TPM, and histopathologic images of disease conditions are shown in **Figures 7B–F**. SG1 emits green-to-blue fluorescence in normal cells and yellow-to-green fluorescence in cancer cells (**Figure 7C**). As the ratio is inverted, SE1 emission color is also reversed (**Figure 7D**). Furthermore, the gland shape was relatively well-maintained in normal but showed an irregular pattern in cancer. This agrees with adenocarcinoma identification confirmed by histopathology after hematoxylin and eosin staining (**Figure 7F**). Therefore, diagnosis using TPM can simultaneously provide three kinds of information: the ratio of value (quantitative profiling), color differentiation, and structural shape, which allows comprehensive and accurate cancer diagnosis.

### Sectional TPM Ratiometric Images for Depth Evaluation

**Figure 8:**
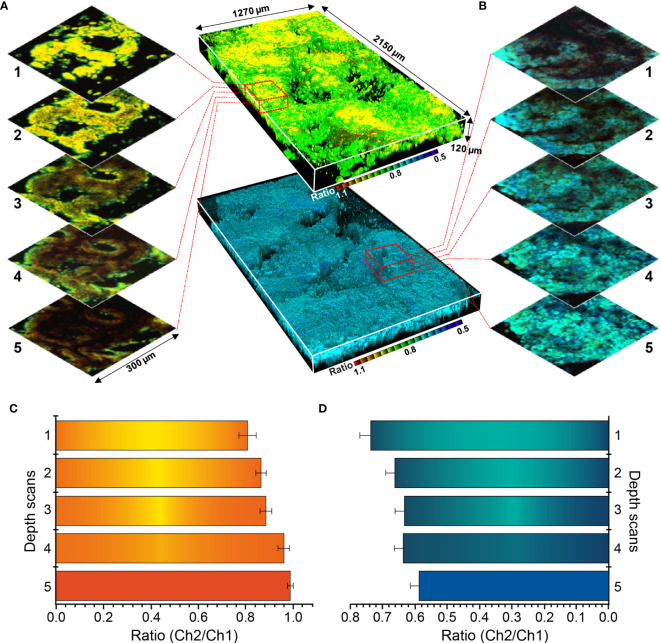
Three-dimensional pseudocolored ratiometric two-photon microscopy images (Ch2/Ch1) of normal and cancer tissue using SE1. **(A)** Sectional images of SE1 in normal tissue. **(B)** Sectional images of SE1 in cancerous tissue. **(C)** Ratio value of each section at varying depths in normal tissue. **(D)** Ratio value of each section at varying depths in cancerous tissue. The red boxed area in panels **(B, C)** are enlarged to show images by depth. Maximum and minimum values are illustrated using error bars.

Sectional images were collected from normal mucosa and cancer lesion, and depth-based imaging was conducted, as shown in **Figure 8**. As the ratio value showed relatively large differences at all depths, SE1 was selected as a probe for depth evaluation. Normal and cancer tissues were observed at approximately 80–200 μm depth and 3D ratiometric fluorescent images were produced using 1700 images taken at each depth. Enzyme-activated fluorescence (observed at the surface) was irregular and disordered, and the ratio values varied according to depth. Thus, changes in color emission enable activation measurement of each detection target.

### Fluorescence Response Over Time for SG1, SE1, and SHC Reactions With Tissue Samples Treated With Lysis Buffer

**Figure 9:**
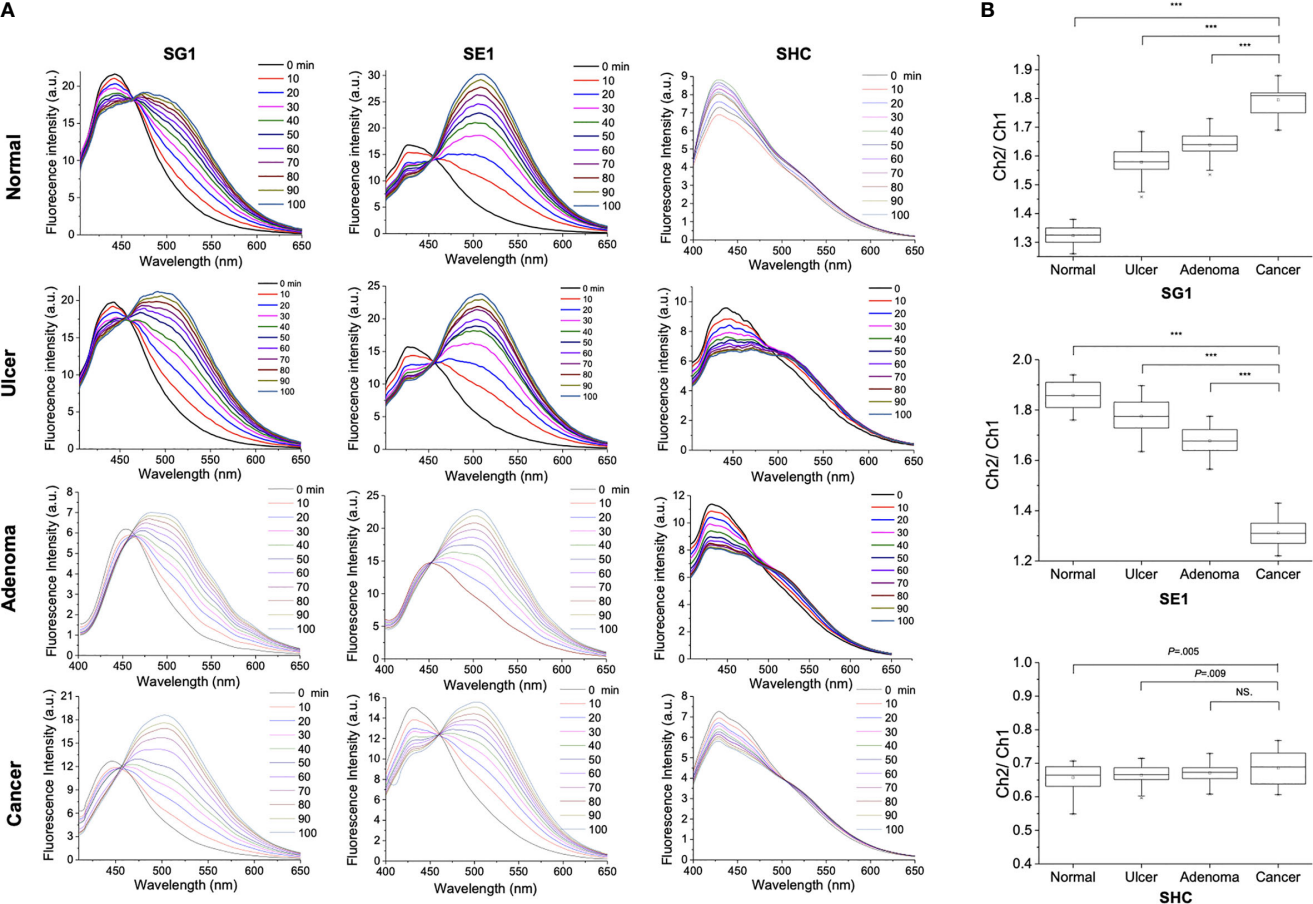
**(A)** Fluorescence response over time for 1 μM SG1, SE1, and SHC reactions with normal and cancerous stomach tissues treated with lysis buffer (T-PER). **(B)** Box plot of the relative ratio value (Ch2/Ch1) from spectra (***P < 0.001, NS, not significant).

**Figure 9** shows the fluorescence response over time for SG1, SE1, and SHC (1 μM) reactions with normal, ulcer, adenoma, and cancer tissue treated with lysis buffer. Galactosidase activity was higher in cancer than in normal (1.794 ± 0.048 *vs*. 1.324 ± 0.035, *P* < 0.001), ulcer (1.579 ± 0.055, *P* < 0.001), and adenoma (1.639 ± 0.047, *P* < 0.001) samples when using SG1. Esterase activity was lower in cancer than in normal (1.859 ± 0.053 *vs*. 1.313 ± 0.051, *P* < 0.001), ulcer (1.775 ± 0.063, *P* < 0.001), and adenoma (1.677 ± 0.051, *P* < 0.001) when using SE1. Conversely, when using SHC, the difference in hNQO1-induced activity between cancer and other conditions was not as prominent as those obtained using SG1 and SE1 (cancer *vs*. normal: 0.687 ± 0.048 and 0.657 ± 0.038, *P* = 0.005, *vs*. ulcer: 0.664 ± 0.029, *P* = 0.009, *vs*. adenoma: 0.671 ± 0.028, *P* = 0.076). These results were consistent with the TPM results, confirming the feasibility of using tissue lysis solution as an effective multi-validation system for cancer diagnosis based on enzymatic activity.

## Discussion

This is the first study to examine the differences between gastric cancer and other benign conditions (normal, ulcer, and adenoma), using various ratiometric TP probes in the acidic stomach environment. Differences in the fluorescence emission of ratiometric probes between normal and cancer samples were observed by TPM, and the feasibility of depth assessment using 3D sectional images was validated. Additionally, the mean ratio value of cancer differed from gastric ulcer and gastric adenoma in SG1 and SE1. Our results showed that TPM could not only serve as an ‘optic biopsy’ but also plays a potential role in determining an appropriate therapeutic method with histopathologic confirmation.

Because it is noninvasive and has a diagnostic time of < 2 h compared to histopathologic confirmation, diagnosis during endoscopy can be conducted in real-time. Based on this, we developed various ratiometric probes and validated their potential for cancer detection in colon tissue (16, 17). However, endoscopic treatment is limited in colon cancer application, as endoscopic resection is recommended only in mucosal cancer or slightly invasive submucosal cancer (23). Although a lesion may be suitable for endoscopic resection, removal is difficult if it is located in a problematic area, such as the hepatic flexure or sigmoid colon. The larger amount of available space in the stomach compared to in the colon made it easier to conduct endoscopic procedures. Therefore, determining a therapeutic method for the stomach in which ESD is the most prevalent treatment will make the role of TPM even more significant. However, the use of probes required for enzymatic reactions in TPM is limited by the acidic stomach environment. The ratio values of SG1 and SE1 showed significant differences between cancer and normal tissues, but clear images of the gland structure could not be obtained as was possible in the colon (16). This issue is likely due to the low stomach pH inducing probe protonation, affecting the enzymatic reaction or partially yielding weak fluorescence. Because visual distinction is important for cancer identification, further studies are needed to improve this technical aspect. Furthermore, probes showing cell toxicity in previous studies (15–17) were stable at 10 μM used in histologic examination in this study.

Gastric cancer can be divided into early gastric cancer and advanced gastric cancer. Endoscopic treatment is recommended for differentiated early gastric cancer that is ≤ 2 cm without ulceration (24). Endoscopic resection is less costly, has a shorter hospitalization period, and is less invasive than surgical resection (25–27), but yields a comparable outcome (28). The prerequisite for endoscopic treatment is complete resection, and the marking process used after the resection margin is decisively important in ESD. If a safety margin is not secured, additional treatment is required after endoscopic resection (29). In this study, the use of ratiometric TP probes allowed both visual and quantitative differentiation between normal and cancer. Additional studies are required to select the most suitable probes for gastric cancer diagnosis and to set cut-off values after data accumulation. Our method can be used for diagnosis and determining therapeutic measures for gastric cancer treatment.

Compared to ultraviolet-vis photons of confocal microscopy, the energy near-infrared photons used in TPM have very low self-absorption, allowing for deeper tissue penetration. This TPM characteristic overcomes the limitations of other approaches that include autofluorescence and the presence of tissue preparation artifacts, allowing for imaging of thick tissue samples and, consequently, extending the viewing area from tissue surfaces to more than 500 μm deep. As a result, TPM can be used to obtain hundreds of cross-sectional images of thick tissue along the z-direction, enabling the production of high-resolution 3D images. Additionally, the energy absorbed by live tissue from the TPM femtosecond pulse laser is much lower than that of the continuous wave laser used in conventional confocal microscopy. Therefore, TPM enables long-term imaging with minimal photobleaching and photodamage (12, 30–32). In the current study, the mucosa portion of the stomach tissue was sampled against the surface of the imaging dish to obtain the whole sectional image with the mucosa shape pressed flatter than it actually was. Therefore, the observed mucosa section is actually smaller *in vivo*. This limitation will be overcome in future studies by changing the tissue sampling system to ensure that a portion of the submucosa is well-fixed in the imaging dish.

Although numerous studies have diagnosed cancer and other diseases using fluorescent probes (33–35), no studies have cross-validated these results using multiple methods. To further demonstrate the reliability of the results obtained from stomach tissues using TPM, we performed fluorescence analysis of the histolytic solution prepared with tissue lysis buffer (T-PER). Unlike the previous method of measuring enzymatic activity through imaging, our method enables the following: 1) an experiment with a probe applied at a consistent concentration for measuring samples; 2) measurement of samples under consistent conditions reflecting various water-soluble and insoluble environments that can exist inside tissues; and 3) simultaneous measurement of multiple samples with a shorter measurement time. However, compared to direct imaging of tissues, this method has the following limitations: 1) the shape of the actual tissue cannot be observed; 2) comparing enzymatic activities and making a diagnosis according to tissue depth is difficult; and 3) source site localization is impossible. However, our approach can be used as a cross-validation method for cancer diagnosis, as it allows for quick and accurate comparative analysis of previous analytic results through imaging.

Traditional histopathologic evaluation takes considerable amount of time as it requires multiple steps to obtain the final result. In addition, it involves the subjective judgment of a pathologist. In this context, we expect that TPM using enzyme-selective fluorescent probes will provide a visual difference and quantitative depth profiling for gastric cancer diagnosis. In this pilot study, we examined whether a quantitative analysis using chemical methods can implement an aspect that traditional medical diagnostic methods have failed at. However, in order to be established as a bedside or primary diagnosis tool, it needs to overcome certain technical hurdles and be applicable to a variety of cases. In particular, given that this pilot study was conducted on biopsy samples, it is necessary to investigate the range in which TPM images can be implemented with specimens of various sizes. In addition, a further study is needed to evaluate the practical use of the technology by comparing the accuracy and consistency between pathologic and TPM results through traditional diagnostic methods on the acquired specimen. Therefore, this pilot study provides a significant contribution to the existing literature by suggesting a candidate probe for gastric cancer diagnosis.

There were some limitations to our study. First, only patients who received histopathologic confirmation of gastric cancer were enrolled in this study. Hence, experiments were conducted in patients known to have cancerous tissue. Second, depth assessment was conducted only in tissues obtained by biopsy sampling. In terms of depth, access is limited to 80–200 μm because of technical limitations. Overcoming this technical limitation through further studies will enable the identification of tumor invasion in deeper areas. Third, probes with strong TP absorption efficiency are needed to image deeper tissue areas. Observing areas with a maximum depth of 500–800 μm will allow accurate tracking of the actual cancer formation location. Finally, the current method involves combining information obtained using different probes after collecting multiple samples from one patient. If a probe that fluoresces at different wavelengths is developed, multiple probes can be simultaneously applied to generate information from multiple fluorescence channels, providing more efficient and accurate results than previous methods.

In summary, an accurate gastric cancer diagnosis is challenging, as it relies on naked-eye evaluation by endoscopists, and histopathologic confirmation is time-consuming. To overcome these limitations, we demonstrated that gastric cancer could be distinguished from normal and benign conditions (ulcer and adenoma) through TPM imaging using the enzymatic activity of ratiometric fluorescent probes. Furthermore, we tried to evaluate the feasibility of mapping cancer using 3D sectional images and show that lesion boundaries and depth could be detected in a noninvasive manner using ratio values and visual information. As this approach is currently in the experimental stage, further studies are required to develop various probes for TPM, which might serve as diagnostic tools for real-time endoscopy, enabling enhanced penetration and accurate image production.

## Data Availability Statement

The raw data supporting the conclusions of this article will be made available by the authors, without undue reservation.

## Ethics Statement

The studies involving human participants were reviewed and approved by Ajou University Hospital Institutional Review Board. The patients/participants provided their written informed consent to participate in this study.

## Author Contributions

C-KN contributed to study concept and design, ethical application, tissue sampling, analysis and interpretation of data, drafting of the manuscript, and critical revision of the manuscript. CL contributed to study concept and design, performing the experiments, data collection, analysis and interpretation of data, drafting of the manuscript, and critical revision of the manuscript. GL contributed to study concept and design, tissue sampling, and interpretation of data. MC and HL contributed to conducting an experiment, interpretation of data, and visualization of the data. JR and YK contributed to diagnosis of pathologic specimens and review of pathologic results. EL and BP contributed to statistical analysis and drafting of the manuscript. HK and SS contributed to study concept and design, analysis and interpretation of data, drafting of the manuscript, and critical revision of the manuscript. All authors contributed to the article and approved the submitted version.

## Funding

HK and CL acknowledge grants from the National Research Foundation of Korea (NRF, 2018R1D1A1B07050835 and 2019R1A2B5B03100278).

## Conflict of Interest

The authors declare that the research was conducted in the absence of any commercial or financial relationships that could be construed as a potential conflict of interest.

## Publisher’s Note

All claims expressed in this article are solely those of the authors and do not necessarily represent those of their affiliated organizations, or those of the publisher, the editors and the reviewers. Any product that may be evaluated in this article, or claim that may be made by its manufacturer, is not guaranteed or endorsed by the publisher.
