# Supplementary material for: A Diagnostic Method for Gastric Cancer Using Two-Photon Microscopy With Enzyme-Selective Fluorescent Probes: A Pilot Study
Source: Front Oncol. 2021 Aug 27;11:634219. doi: 10.3389/fonc.2021.634219 (PMC8429903; doi:10.3389/fonc.2021.634219)
Supplement: Supplementary file 1 [file DataSheet_1.docx]

Supplementary Material

**Supplementary Method**

# Western blot analysis

Tissue protein (10 μg) was electrophoresed on SDS–PAGE gels and then transferred to PVDF membranes using a wet transfer system (Bio-Rad Laboratories, Hercules, CA, USA). The membrane was incubated with 5% non-fat dry milk in TBST (10 mM Tris–Cl, pH 8.0, 150 mM NaCl and 0.1% Tween-20 [v/v]) at room temperature for 1 h and then probed with the appropriate primary antibodies. Primary antibodies were used for the detection of β-gal (Cell signaling Technology, Danvers, MA, USA), CES (Santa Cruz Biotechnology, Santa Cruz, CA, USA), and hNQO1 (Santa Cruz Biotechnology, Santa Cruz, CA, USA). The immunoreactions were detected by HRP-conjugated secondary antibodies. Visualization was accomplished using the Clarity™ Western ECL substrate (Bio-Rad Laboratories, Hercules, CA, USA) and ChemiDoc™ (Bio-Rad Laboratories, Hercules, CA, USA).


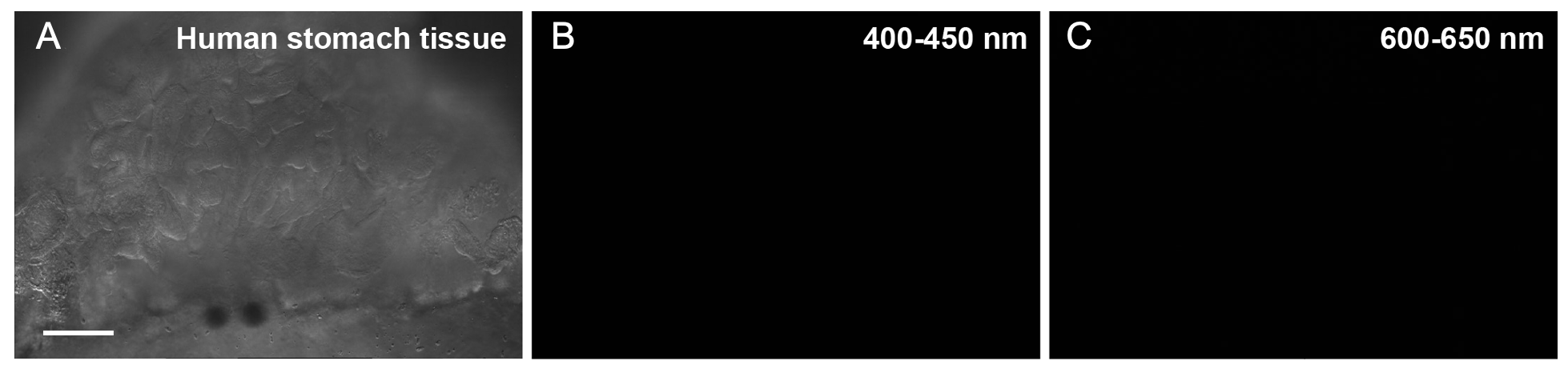


**Supplementary Figure S1.** TPM images of human stomach normal tissue without incubation of probes. (A) Bright-field images of the stomach tissue. (B, C) TPM images of tissue were collected at (B) 400-450 nm and (C) 600-650 nm upon excitation at 750 nm with a femtosecond pulse, respectively. Scale bars: 400 μm.

**
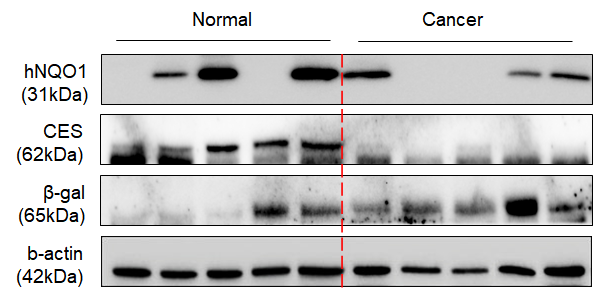
**

**Supplementary Figure S2.** Expression of candidate three enzymes using western blot.
